# Multifunctional Smart ZnSe-Nanostructure-Based Fluorescent Aptasensor for the Detection of Ochratoxin A

**DOI:** 10.3390/bios12100844

**Published:** 2022-10-08

**Authors:** Muhammad Azhar Hayat Nawaz, Muhammad Waseem Fazal, Naeem Akhtar, Mian Hasnain Nawaz, Akhtar Hayat, Cong Yu

**Affiliations:** 1 State Key Laboratory of Electroanalytical Chemistry, Changchun Institute of Applied Chemistry, Chinese Academy of Sciences, Changchun 130022, China; 2 University of Science and Technology of China, Hefei 230026, China; 3 Interdisciplinary Research Centre in Biomedical Materials (IRCBM), COMSATS University Islamabad, Lahore Campus, Lahore 54000, Pakistan; 4 Institute of Chemical Sciences, Bahauddin Zakariya University (BZU), Multan 60800, Pakistan

**Keywords:** Förster resonance energy transfer, ZnSe nanostructures, ochratoxin A, aptamer, zeta potential, fluorescence detection

## Abstract

Herein, we present a comprehensive investigation of rationally designed zinc selenide (ZnSe) nanostructures to achieve highly negatively charged ZnSe nanostructures. A Microwave-assisted hydrothermal synthesis method was used to synthesize three types of ZnSe nanostructures, i.e., nanorods, µ-spheres and nanoclusters, as characterized by a zeta potential analyzer, X-ray diffraction (XRD), scanning electron microscopy (SEM), Raman spectroscopy and BET, which were labeled as type A, B and C. Three different solvents were used for the synthesis of type A, B and C ZnSe nanostructures, keeping other synthesis conditions such as temperature, pressure and precursors ratio constant. Based on two heating time intervals, 6 and 9 h, types A, B and C were further divided into types A6, A9, B6, B9, C6 and C9. ZnSe nanostructures were further evaluated based on their fluorescent quenching efficiency. The maximum fluorescence quenching effect was exhibited by the ZnSe-B6 type, which can be attributed to its highly negative surface charge that favored its strong interaction with cationic dye Rhodamine B (Rh-B). Further, the optimized ZnSe-B6 was used to fabricate an aptasensor for the detection of a food-based toxin, ochratoxin-A (OTA). The developed aptasensor exhibited a limit of detection of 0.07 ng/L with a wide linear range of 0.1 to 200 ng/L.

## 1. Introduction

The development of biosensing devices that can monitor environmental and dietary hazards has grabbed much attention in recent years [1,2]. In particular, fluorescent biosensors based on bio-conjugated nanomaterials and involving a Förster resonance energy transfer (FRET) mechanism have great advantages in term of sensitivity and simple operating procedures [3]. The choice of materials used to develop fluorescent biosensors is very crucial because surface charge, electron transport behavior and structural and optical properties decide the ability of materials to perform selective, sensitive and reliable detection [3]. Thus, there is a dire need of such materials in which controlling their synthesis parameters can induce different properties and functionalities such as surface charge and morphology. Recently, diverse types of nanomaterials such as carbon nanotubes, 2D nanomaterials, gold nanoparticles and quantum dots have been explored and investigated as potential fluorescent quenchers for the development of fluorescent biosensors [4].

Zn-based II–VI nanostructures such as zinc selenide (ZnSe) exhibit wide direct bandgap (2.67 eV) along with high exciton binding energy (21 meV); thus, ZnSe has been considered as an excellent choice for optoelectronic devices [5,6,7]. In recent times, researchers working in the domain of nanostructured materials have reported a number of different dimensional ZnSe nanostructures including nanorods, nanoplates, nanoneedles, nanobelts, nanoparticles and nanowires [8,9,10,11,12,13,14]. Diverse approaches have been applied for the synthesis of ZnSe nanostructures, such as microwave irradiation, hydrothermal, chemical vapor deposition and solvothermal [15,16,17,18]. Among these, the microwave-assisted synthesis technique has several advantages such as fast and uniform heating, higher product yield and short reaction time, low energy requirement, less expensive, high purity and small narrow particle size distribution as compared to other techniques [19]. As the fluorescent sensing performance is directly linked to surface charge, size, dispersity and morphology of nanostructures materials, thus the synthesis of ZnSe nanostructures with controlled surface charges, morphology, and physicochemical properties has great potential for biosensing applications [20,21,22,23].

Here, we show that by varying the synthesis reaction time and changing precursors, ZnSe nanostructures with different morphology, size, surface charge and physicochemical properties can be designed for fluorescent detection applications. The microwave-assisted synthesis route was used to synthesize three types of ZnSe nanostructures by changing their precursors and reaction time (6 and 9 h) at fixed temperatures. Based on synthesis conditions, three different shapes including spherical, rods and cluster-like morphologies were formed. XRD results of synthesized ZnSe nanostructures revealed through Williamson–Hall equations that nanostructures contain strains. It is well-established that due to the strain, defects are produced, which strongly influence the intrinsic properties [24] of synthesized ZnSe, resulting in improvements to their dispersion [25]. At the same time, defects induce negative charge; thus, the surface of the ZnSe nanostructures exhibited highly negative surface charges, which were confirmed by measuring their zeta potentials [26]. This highly negative surface charge made the synthesized ZnSe nanostructures highly stable and dispersive [27,28]. With the increase in size, the negative surface charge also increased, which may be ascribed to a larger steric hindrance [29]. The highly negative surface charges of ZnSe nanostructures were further exploited to interact with Rh-B a cationic dye and their behavior as nanoquenchers was confirmed. Ochratoxin A (OTA) is a mycotoxin classified as a (Group-2B) carcinogen by the International Agency for Research on Cancer (IARC). OTA released in the form of Aspergillus and Penicillium results in the contamination of foods such cereals, fruit juices, beans, wine, corn, wheat and barley [30]. The European Commission has released guidelines about the permittable content of OTA, which is 5 µg/kg and 10 µg/kg in raw grains and soluble coffee and 2 µg/kg in grape juice or wine [31].

**Scheme 1:**
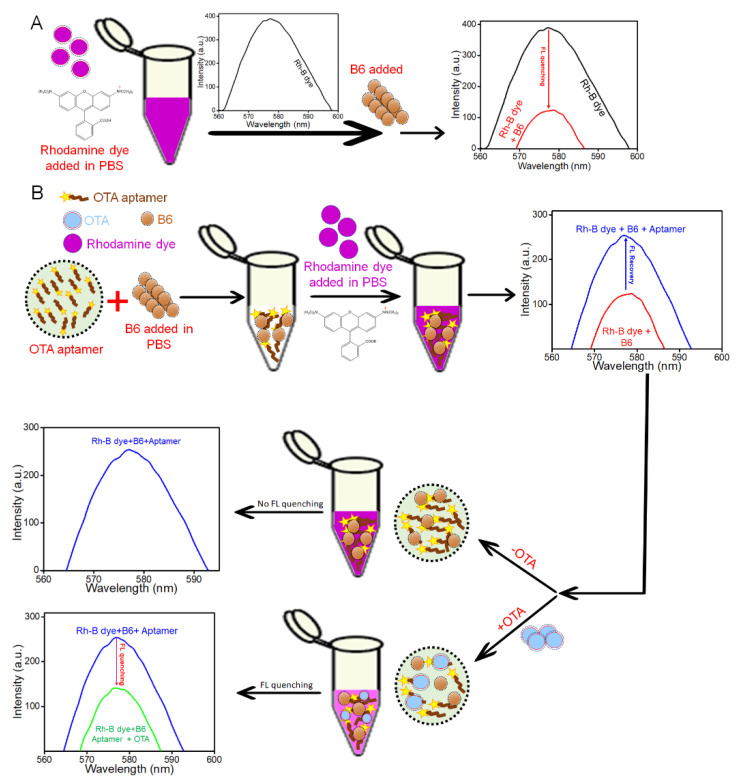
Schematic illustration of the fluorescence-quenching-based detection of OTA; quenching response of ZnSe-B6 on fluorescence emission spectra of Rh-B dye (**A**), quenching response of ZnSe-B6 on fluorescence emission spectra of Rh-B dye in the presence of OTA aptamer with and without OTA target (**B**).

ZnSe nanostructures were exploited as nanoquenchers to develop the fluorescent aptasensor for the detection of OTA. The quenching response of type ZnSe-B6 on the emission spectrum of Rh-B dye in the presence of an OTA aptamer was evaluated with and without an OTA target. Pure Rh-B exhibited a sharp fluorescence peak, which significantly reduced after the addition of ZnSe-B6 nanoquencher, which primarily can be attributed to an FRET mechanism based on the strong binding interaction between cationic Rh-B dye and the highly negatively charged surface of ZnSe-B6 [32,33]. Scheme 1B shows step by step details of the fabrication of a ZnSe-nanostructure-based fluorescent aptasensor for the detection of OTA. Firstly, exploiting very high negative surface charge of ZnSe-B6 and positive charge of amino modified OTA aptamer, the ZnSe-B6–aptamer complex was formed. Afterwards, when Rh-B dye was introduced to the ZnSe-B6–aptamer complex, fewer ZnSe-B6 particles were available to quench the fluorescence of the Rh-B dye, and thus a fluorescence recovery response was achieved. Finally, when target analyte (OTA) was introduced, it made specific interactions with the aptamer, which weakened aptamer and ZnSe-B6 interactions. The stronger conjugation between the amino-modified OTA aptamer and target analyte OTA resulted in ZnSe-B6 particles being released from the ZnSe-B6–aptamer complex, which quenched the fluorescence of the Rh-B dye, and thus fluorescence quenching recovered. There was a direct proportional relation between the fluorescence quenching percent and the concentration of OTA in the assay. In this work, based on synthesis reaction time and precursors, we have rationally designed ZnSe nanostructures with highly negative surface charges and different morphologies and exploited these properties to develop an aptasensor for OTA detection.

## 2. Experimental

### 2.1. Synthesis of ZnSe Nanostructures

A microwave-assisted hydrothermal synthesis approach was used to synthesize three types of ZnSe nanostructures under control synthesis conditions by keeping temperature, pressure and precursors ratio constant by using three different solvents at two heating time intervals, including 6 and 9 h. Under controlled synthesis conditions, three different shapes, including spherical, rods and cluster-like morphologies, were obtained at two different time intervals. Brief detail about the synthesis of these morphologies is as follows.

#### 2.1.1. Fabrication of ZnSe Nanorods

For the preparation of ZnSe nanorods, 1.6 mmol of zinc nitrate was mixed with 4 M KOH in 30 mL (water/ethanol) and kept heating at 80 °C in a microwave oven for 20 min. After that, 1.5 mmol of selenium tetrachloride in 20 mL hydrazine dihydrochloride was added drop wise into the previous solution and this reaction was kept for 6 and 9 h at 120 °C in a microwave-assisted oven to obtain two types of ZnSe, which were labeled as A6 and A9 respectively. The resultant precipitates of A6 and A9 were cooled down to room temperature and then centrifuged and washed with water and ethanol, respectively. The obtained powder was next dried at vacuum in room temperature.

#### 2.1.2. Fabrication of ZnSe µ-Spheres

Similarly, for the preparation of ZnSe µ-spheres, 1.6 mmol of zinc nitrate was mixed with 4 M KOH in 30 mL (water/ethanol) and kept heating at 80 °C in a microwave oven for 20 min. After that, 1.5 mmol of selenium tetrachloride in 20 mL ethylene glycol was added drop wise into the previous solution and this reaction was kept for 6 and 9 h at 120 °C in a microwave-assisted oven. The materials kept for 6 and 9 h were labeled as B6 and B9, respectively. The resultant precipitate was cooled down at room temperature and centrifuged and washed with water and ethanol and subjected to drying in a vacuum oven.

#### 2.1.3. Fabrication of ZnSe Nanoclusters

Further, for the preparation of ZnSe nanoclusters, 1.6 mmol of zinc nitrate was mixed with 4 M KOH in 30 mL (water/ethanol) and kept under microwave in an oven for 20 min at 80 °C. In the next step, 1.5 mmol of selenium tetrachloride was prepared in 20 mL acetic acid and was added drop wise into the previous solution and this reaction was kept for 6 and 9 h at 120 °C in a microwave-assisted oven. After that, the solution was placed into a microwave oven for 6 and 9 h to obtain C6 and C9, respectively. The resultant precipitate was cooled down at room temperature and then centrifuged and washed with water and ethanol, respectively. The resultant precipitates were next dried in a vacuum oven.

### 2.2. ZnSe-Nanostructure-Based Fluorescence Quenching

Blank and fluorescence quenching measurements of organic fluorescence dye Rh-B were carried out without and with the addition of ZnSe nanoquenchers, respectively. First, a blank reagent fluorescence emission spectrum containing (160 ng/L) dye was measured. Afterwards, 1 µL from each stock solution containing ZnSe nanostructures (A6, A9, B6, B9, C6, C9) were added into Rh-B (16 µL) and a volume make up of 2000 µL was achieved using PBS to get an end concentration of 450 µg/L for ZnSe nanostructures. Subsequently, the mixture stayed for 10 min and then fluorescence quenching on the addition of all six ZnSe nanostructures was determined using 554 nm as the excitation wavelength while emission spectra were noted at 578 nm. The extent of fluorescence quenching was measured by calculating the difference between the mission spectra of the Rh-B and the maximum of emission induced by the ZnSe nanostructures. The maximum quenching was observed by the ZnSe nanostructure type B6, and thus used in the next experiments to develop the aptasensor for OTA detection. A 2000 µL reaction volume with an optimum concentration of 450 µg/L ZnSe was used for the experiments to detect OTA.

### 2.3. ZnSe-Nanostructure-Based Fluorescent Aptasensor for OTA Detection

The optimized amino-modified OTA aptamer (50 nM) was incubated with 1 µL ZnSe type B6 (450 µg/L) for 15 min. Afterwards, a total volume of 2000 μL of the solution containing aptamer, ZnSe-B6 and dye Rh-B (160 ng/L) was made using PBS and further incubated for 10 min. Subsequently, the solution was subjected to fluorescence response and then ultimately varying concentrations of OTA (0.1–200 ng/L) were added. An optimized incubation time, i.e., 30 min, was used to incubate the OTA containing solution and then fluorescence measurements were taken at 554 nm for excitation and 578 nm for emission. The control experiment was also run, without the addition of OTA.

### 2.4. Interference and Real Sample Studies

The specificity of the developed OTA aptasensor was evaluated in the presence of the possible interfering analytes, i.e., ochratoxin-B, aflatoxin M1 and aflatoxin B1. The same concentration (100 ng/L) of each interfering species was employed to form an aptamer-interfering species complex using the same parameters as those applied for OTA detection. Further, a rice sample purchased from the local market of Lahore, Punjab and grinded in a kitchen grinder machine was used to confirm the practical acceptability of the fabricated aptasensor. The rice extract was received following the already reported procedure [34]. Acetonitrile was used as an extracting solvent and one gram of grinded rice powder was introduced into a 10 mL solvent, and stirred for 15 min. Afterwards, a 15 min centrifugation of the mixture was carried out at 35,000 rpm, and then the received solid rice sample was dried under an inert atmosphere. For real sample validation, the rice extract and PBS (1:9 *v*/*v*), having a total volume of 1 mL, was firstly sonicated to get a well-mixed solution. Subsequently, 1 µL of ZnSe-B6 (450 µg/L), and for 50 nM end concentration, 20 µL of amino-modified OTA aptamer, were used for the fabrication of an aptasensor. Percentages of quenching recoveries with OTA-spiked (5, 50 and 100) ng/L rice samples were determined, which presented excellent linear behavior with the increase of OTA concentration.

## 3. Results and Discussion

### 3.1. Morphological and Structural Characterization of ZnSe Nanostructures

**Figure 1:**
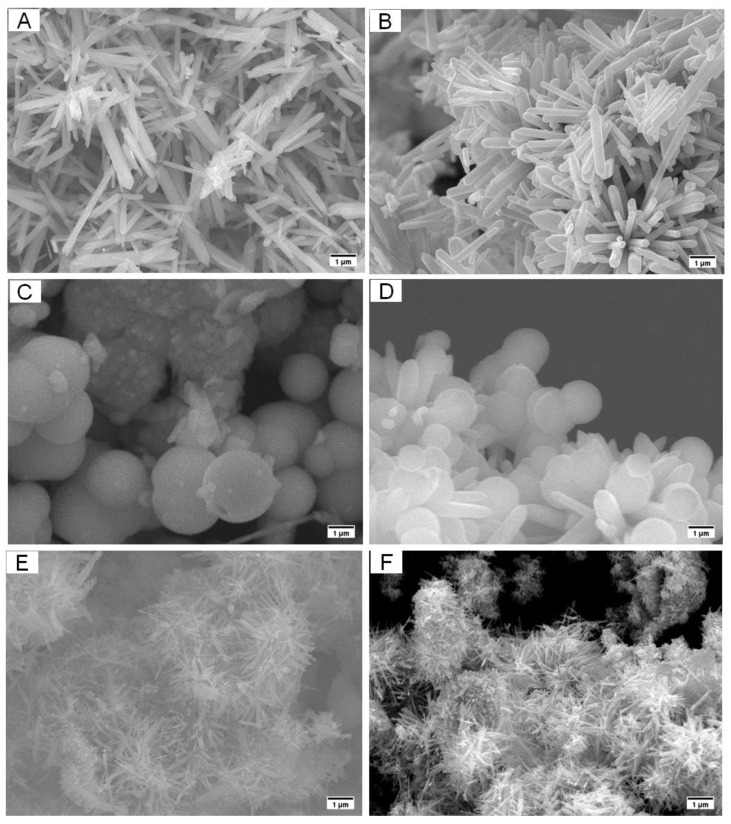
SEM images of ZnSe nanostructures: ZnSe nanorods (**A**,**B**), nanospheres (**C**,**D**) and nanoclusters (**E**,**F**).

The surface morphologies of the synthesized ZnSe nanostructures were characterized by scanning electrode microscopy (SEM). SEM results showed the formation of ZnSe with different shapes and sizes. Briefly, Figure 1A and B shows the clear formation of nanorod shaped ZnSe with an average width of 200–260 and 150–210 nm, respectively. Similarly, Figure 1C and D clearly shows the successful formation of ZnSe µ-spheres with an average size of 2200–2400 and 1100–1500 nm, respectively. Further, Figure 1E and F shows the formation of ZnSe nanoclusters with an average size of 210–260 and 75–130 nm, respectively.

**Figure 2:**
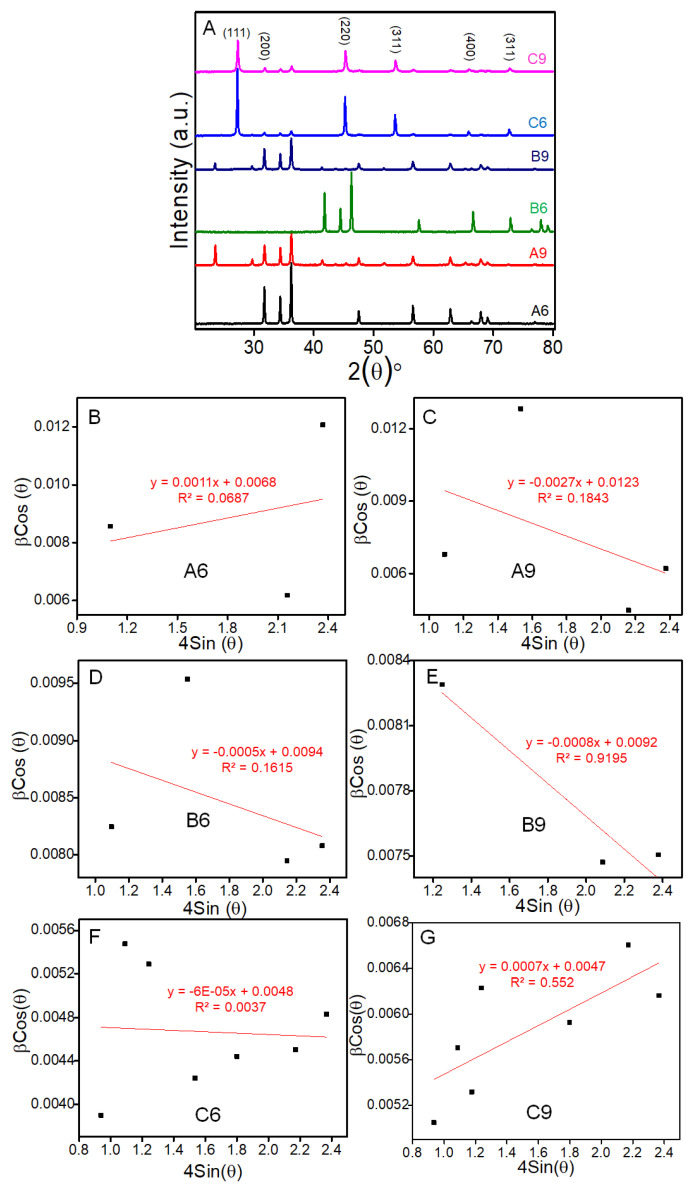
XRD Spectra representing formation of ZnSe nanostructures (**A**), and the crystallite size and strain of synthesized ZnSe calculated by using the W–H plot (**B**–**G**).

The phase purity, crystallographic structure and crystal formation of zinc selenide was investigated via wide-angle XRD analysis within the range of 5–80° (Figure 2A).

The XRD spectrum reflects a series of diffraction characteristic peaks centered at 2θ value of 26.99°, 31.55°, 44.55°, 53.45°, 65.78° and 72.48° corresponding to 111, 200, 220, 311, 400 and 331 crystal planes of face-centered cubic, respectively. These results clearly match with the Joint Committee on Powder Diffraction Standard (JCPDS No. 88-2345), thus confirming the formation of ZnSe.

Further, the average crystallite size and strain of synthesized ZnSe nanostructures were also calculated by using Scherrer (Supplementary Equation (S1)) and Williamson–Hall equations, respectively. The calculated average crystallite sizes of A6, A9, B6, B9, C6 and C9 were observed to be 16.69, 21.14, 16.48, 17.91, 30.05 and 23.91 nm, respectively (Figure 2B–G and Supplementary Table S1). The micro strain was also calculated by using the Williamson–Hall equation. From the results, two types of micro strains were observed in which positive slopes of 1.1 and 0.7 for A6 and C9 showed a tensile strain, whereas negative slopes of −2.7, −0.5, −0.8 and −0.06 for A9, B6, B9 and C6 showed a compressive strain, respectively [34].

**Figure 3:**
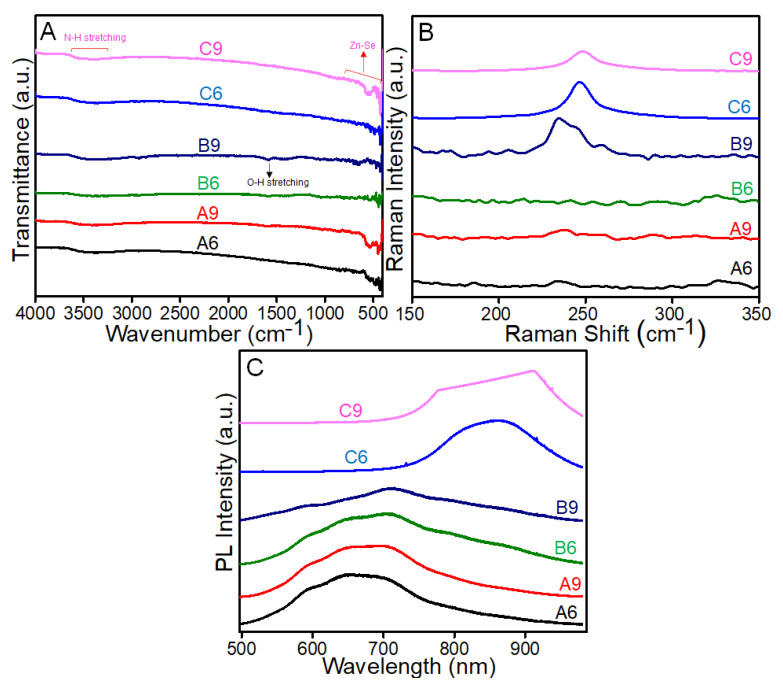
FTIR spectra of ZnSe nanostructures (**A**), Raman spectra (**B**) and PL spectra (**C**) of synthesized ZnSe nanostructures.

The bending, stretching, rotational and vibrational modes in the synthesized ZnSe nanoparticle were studied via Fourier transform infrared spectroscopy (FTIR). FTIR spectra of synthesized ZnSe showed the characteristic peaks at 482, 561, 651, 671 and 970 cm^−1^ belonged to Zn-Se vibrations (Figure 3A). In addition, the characteristics peak at 3429 cm-1 and weak characteristics peak at 1595 cm^−1^ corresponded to O-H characteristic vibrations. In the case of C2 and C3, there was another sharp peak at 3199 cm^−1^, which corresponded to the N-H stretching vibration band. Additionally, a slight shift of the N-H stretching vibration band toward the lower frequency could be attributed to the interaction of N_2_H_4_ with zinc ion and the regular periodic structure of the molecular precursor.

Furthermore, Raman (Figure 3B) and PL (Figure 3C) spectroscopy were also used to analyze the synthesized ZnSe nanostructures. Raman results showed the presence of TO (210 cm^−1^) and LO (255 cm^−1^) phonon frequencies for bulk ZnSe [35]. The transverse optic (TO) and longitudinal optic (LO) phonon modes of the crystalline ZnSe were responsible for the two Raman peaks with centers at 205 and 248 cm^−1^, respectively. Results further showed that the synthesized ZnSe structures were of excellent crystalline quality and pure phase, as seen by the crisp and symmetrical Raman peaks. However, the TO and LO phonon frequencies for ZnSe nanostructures were centered at 199 and 242 cm^−1^, respectively, and both yielded a wide Raman peak as a result of the structures’ high surface-to-volume ratio.

PL spectra of ZnSe nanostructures revealed a strong and wide emission band spanning 500–800 nm. The near-band-edge (NBE) emission of ZnSe is often responsible for the faint blue emission peak. Surface emissions and potential metal vacancies have been linked to the emission band between 520 and 780 nm in the case of A6, A9, B2 and B9. According to Geng et al. [36], certain donor–acceptor pairings connected to Zn vacancy and interstitial states, or linked to dislocation stacking faults and nonstoichiometric defects, are the reason behind the high emission at about 520 nm. Whereas, according to Zhang et al. [37], recombination of a donor–acceptor pair involving Zn vacancies and Zn interstitial was the cause of the emission. Thus, we believe that the high emission must be related to the interstitial Zn defect and nonstoichiometric defects since the products increased under Zn-rich conditions in the case of C6 and C9. Brunauer−Emmett−Teller (BET) has been used to characterize the surface area and porous texture of ZnSe. BET results showed specific surface areas of ZnSe of 0.004, 0.048, 0.016, 0.020, 0.016 and 0.014 m^2^/g, corresponding to A6, A9, B6, B9, C6 and C9, respectively. The zeta potential of ZnSe nanostructures were also calculated to estimate the surface charge. All the ZnSe nanostructures exhibited higher values of negative zeta potential (Table 1). The negative zeta potential value for the synthesized ZnSe may be attributed to the dense electron of O around ZnSe. It can be seen in Table 1 that ZnSe-B6 (µ-sphere) had the highest negative zeta potential value, which makes ZnSe-B6 highly dispersive and stable.

### 3.2. Morphological-Based Fluorescence Quenching of the ZnSe Nanostructures

**Figure 4:**
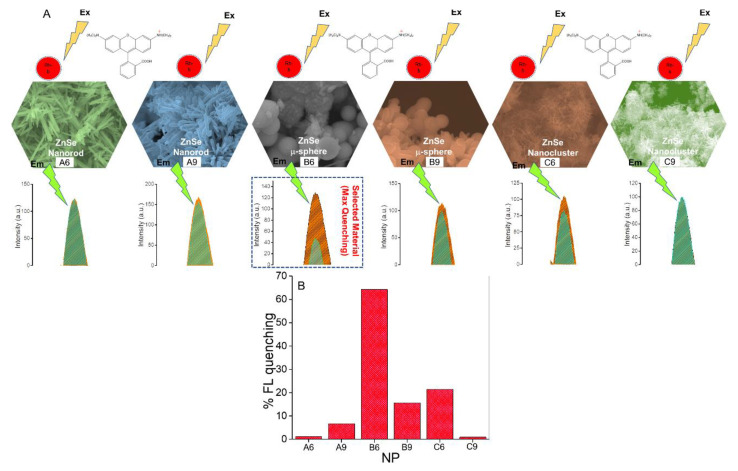
Summary of fluorescence quenching behavior of ZnSe nanostructures (A6–C9) (450 µg/L) based on structural morphology and negative surface charge (Rh-B: 160 ng/L) (**A**). % fluorescence quenching effect of ZnSe (A6, A9, B6, B9, C6, C9) (**B**).

**Figure 5:**
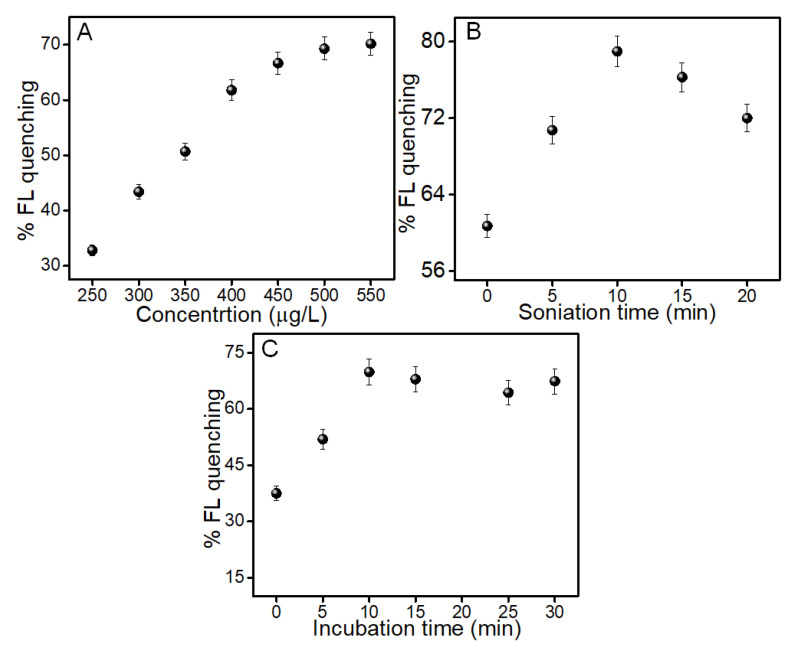
Effects of ZnSe-B6 concentrations (250–550 µg/L) (**A**), sonication time (**B**) and incubation (**C**) on the quenching (% FL) in the presence of Rh-B (160 ng/L).

Figure 4A shows the summary of fluorescence quenching behavior of ZnSe nanostructures (A6–C9) based on negative surface charge, size and morphology. The highest negative value of zeta potential exhibited by ZnSe-B6 (µ-sphere) showed a maximum %FL quenching signal because of extensive surface interaction with cationic dye Rh-B. Figure 4B shows comparative % fluorescence quenching behavior of all six ZnSe (A6–C9) nanostructures. Rh-B concentrations in the range of 10–250 ng/L were also optimized (Supplementary Materials, Figure S1) for the development of a ZnSe-nanostructure-based aptasensor. We further evaluated the effect of ZnSe-B6 concentration (Figure 5A) and found small difference of concentrations values greater than 450 µg/L, and thus opted for the development of an aptasensor. Next, sonication conditions and the incubation time were also employed to adjust the fluorescence measurements (Figure 5B,C). A 10 min sonication time and a 10 min incubation time were selected as optimized values for the next experiments.

### 3.3. Fabrication of the ZnSe-Based Aptasensor

To fabricate the ZnSe-nanostructure-based aptasensor, exploiting their quenching properties, the quenching response of selected ZnSe-B6 on fluorescence emission spectra of Rh-B dye in the presence of an OTA aptamer was evaluated with and without OTA target, as shown in Scheme 1.

**Figure 6:**
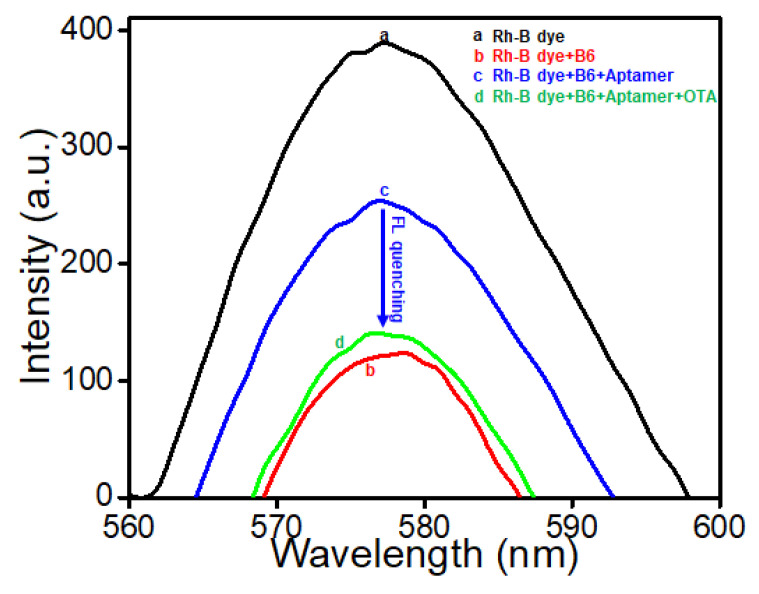
Fluorescence emission spectra of Rh-B (160 ng/L) (**a**); quenched fluorescence emission with ZnSe-B6 nanoquencher of 450 µg/L (**b**); fluorescence recovery with OTA aptamer (50 nM) stabilized with (450 µg/L) ZnSe-B6 (**c**); fluorescence quenching recovery with OTA (**d**).

As shown in Figure 6a, the pure dye solution (160 ng/L) presented a strong fluorescence maximum at 578 nm. On adding a ZnSe-B6 nanoquencher, an appreciable decrease in fluorescence response was found (Figure 6b), which is primarily can be attributed to Förster resonance energy transfer (FRET), based on the strong binding interaction between cationic Rh-B dye and the highly negatively charged surface of ZnSe-B6 [32,33]. To develop the ZnSe-B6-based aptasensor, already optimized concentration, sonication time and incubation time values for the ZnSe-B6 were used. ZnSe-B6 (450 µg/L) and 50 nM amino-modified aptamer was chosen for the ZnSe-B6–aptamer complex formation. ZnSe-B6 exhibited very high negative surface charge while the amino-modified OTA aptamer contained a positive charge due to the attached amino group, which favored their surface interactions. The solution mixture was subjected to incubation for 15 min to enhance the surface interaction between ZnSe–aptamer conjugations.

Subsequently, when Rh-B dye was introduced to the ZnSe-B6–aptamer conjugation, a fluorescence recovery response was achieved (Figure 6c). Upon introduction of the OTA, conjugation between the aptamer and OTA formed, which weakened the link between the aptamer and ZnSe-B6. The OTA and aptamer’s stronger binding interaction resulted in the release of ZnSe-B6 particles from the ZnSe-B6–aptamer complex, which quenched the fluorescence of the Rh-B dye, and thus fluorescence quenching recovered (Figure 6c). There was a direct proportional relation between the fluorescence quenching percent and the concentration of OTA in the assay.

### 3.4. Optimization

**Figure 7:**
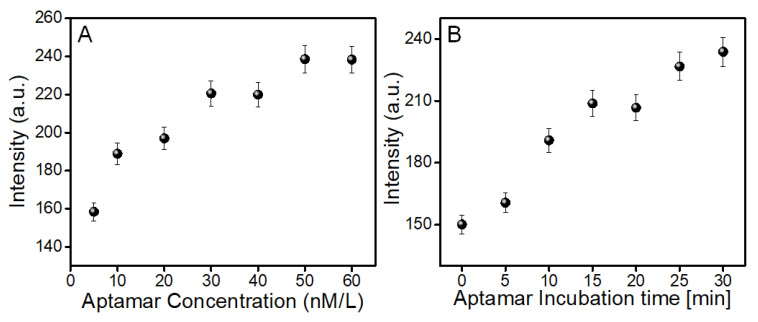
Effect of aptamer concentrations (5 to 60 nM) (**A**) and aptamer incubation time (0–30 min) (**B**) on fluorescence recovery of Rh-B (160 ng/L) in the presence of ZnSe-B6 (450 µg/L).

**Figure 8:**
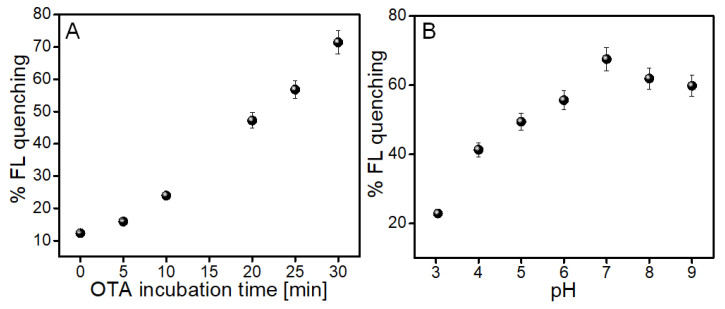
Effect of incubation time (0–30 min) of OTA (200 ng/L) (**A**) and pH (3–9) on % Fl quenching (**B**).

After demonstration of the quenching properties of the ZnSe-B6 to develop the OTA aptasensor, the next experiments were carried out to fix the experimental conditions, including aptamer concentration, aptamer incubation time, pH and incubation time of OTA, to evaluate their influence on the aptasensor efficiency. Figure 7A demonstrates the influence of different concentrations of OTA aptamer (5–60 nM), which demonstrated an incremental fluorescence intensity with increases in the amount of aptamer. Based on this observation, a 50 nM concentration was selected to fabricate the aptasensor, as this concentration was appropriate to devise the aptasensor based on quenching and recovery signals. Figure 7B showed that with increases in incubation time (0–30 min), an increase in the fluorescence emission also took place. An aptamer incubation time of 15 min was selected for the development of the aptasensor. The effect of incubation time of OTA (0–30 min) on fluorescence quenching recovery and performance of the aptasensor was further assessed. Figure 8A shows that maximum fluorescence quenching (% FL quenched) after OTA target conjugation with the aptamer complex took place after 30 min incubation. The fluorescence quenching on introduction of OTA at varying pH levels (pH 3–9) was also evaluated and the quenching response has been given in Figure 8B. An impressive %FL quenching response of ~70% was observed at 7 pH.

### 3.5. Analytical Performance of the Developed Aptasensor

**Figure 9:**
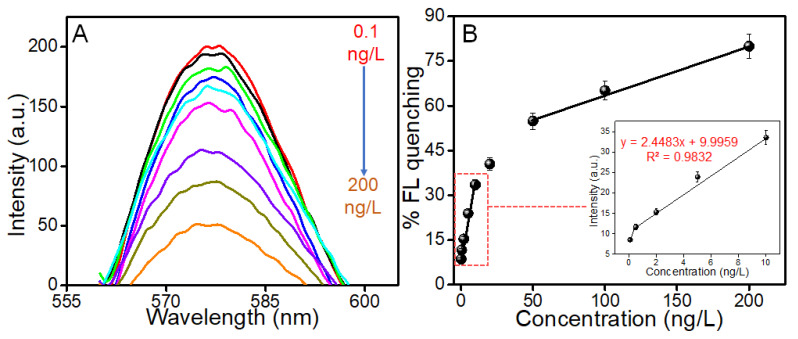
Fluorescence quenching spectra at different OTA concentrations (**A**); calibration plot (%FL quenching vs. OTA concentrations) (0.1, 0.5, 2.0, 5.0, 10, 20, 50, 100 and 200 ng/L) measured at 554 nm for excitation and 578 nm for emission (**B**).

To confirm the practical applicability of the fabricated ZnSe-nanostructure-based aptasensor, the calibration curve based on % of recovered FL quenching with OTA concentrations ranging from 0.1–200 ng/L was evaluated. It can be seen in Figure 9. The %FL quenching signal increased in a directly proportional manner with higher concentration values of OTA due to the strong binding coordination of the amino-modified OTA aptamer with OTA to form an OTA aptamer–OTA reaction complex. Figure 9B shows that there is a good linear relationship, which can be confirmed with a linear equation (y = 2.4x + 9.99, R^2^ = 0.983) between recovered %FL quenching and OTA concentration. A very low limit of detection (LOD) of 0.07 ng/L was achieved. The low LOD is an indication of the high specificity and selectivity of the ZnSe nanostructure-based aptasensing system.

**Figure 10:**
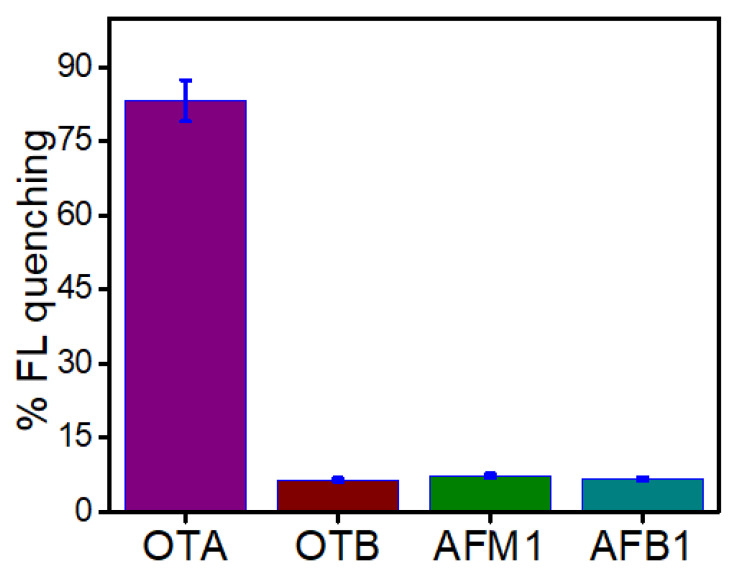
Response of developed aptasensor for OTA and other interfering species at a concentration of 200 ng/L in PBS (pH 7) under optimized conditions.

The complex composition of samples is a big challenge for the development of aptasensors; thus, selectivity is the most important factor towards the practical application of aptasensors [34]. Selectivity of the ZnSe-nanostructure-based fluorescent aptasensor towards OTA was assessed using OTA interfering species such as ochratoxin-B (OTB), aflatoxin-M1 and aflatoxin-B1 (AFB1). Firstly, %FL quenching recovery was evaluated in the presence of 200 ng/L OTA. Afterwards, the relative %FL quenching recovery was evaluated in the presence of some common OTA interfering species, each at a concentration of 200 ng/L, under the same experimental parameters as those followed for OTA. Figure 10 shows a comparative % recovered FL quenching response of interfering compounds, which suggests that all interfering mycotoxins exhibited very little %FL quenching recovery as compared to OTA, which is a clear indication that there is negligible interaction between the OTA aptamer and the interfering analytes. The relative perecntages of recovered FL quenching were ~83% for OTA, ~6.51% for OTB, ~7.38 for AFM1 and ~6.59% for AFB1. The results demonstrate high selectivity of the developed aptasensor, which is due to the high specificity interaction and binding of the OTA with the aptamer [38,39].

The analytical accuracy, practical applicability, reliability and reproducibility of the ZnSe-nanostructure-based fluorescent aptasensor was validated by detecting OTA in rice extracts. Three varying concentrations of OTA, i.e., 5, 50 and 100 ng/L were spiked to rice samples purchased from a local market. The recovery of spiked OTA was determined using the developed aptasensor. As shown in Table 2, the recovery and reproducibility were acceptable in all cases. The FL quenching recovery % response of OTA in buffer was in strong agreement with OTA spiked samples. The relative standard deviation (R.S.D %) of these recovery experiments were 0.56%, 0.22% and 0.218% for n = 3, which demonstrates good stability, reproducibility and practical applicability of the developed aptasensor. Comparison of some recent nanostructure-based fluorescent sensors for OTA detection is presented in Supplementary Table S2.

## 4. Conclusions

In the present work, precursor and synthesis reaction time dependent multifunctional ZnSe nanostructures, with a highly negative surface charge and different sizes and morphologies were successfully synthesized. We further exploited ZnSe µ-sphere nanoquenchers for the development of a fluorescent aptasensor for the detection of ochratoxin A. Fluorescence results confirmed that ZnSe µ-spheres had maximum quenching efficiency as compared to other types of synthesized ZnSe nanostructures, because of their highly negative surface charge and large size. Highly dispersive, stable and negatively charged ZnSe µ-spheres exhibited strong interaction with cationic Rh-B dye. Similarly, amino-modified OTA aptamer also strongly interacted with ZnSe µ-spheres. Exploiting these interactions, a ZnSe-µ-sphere-based fluorescent aptasensor was successfully fabricated for the detection of ochratoxin A. The proposed OTA aptasensor demonstrated a low detection limit of 0.07 ng/L with an excellent wide linear range of 0.1 to 200 ng/L. Moreover, we also evaluated the practical applicability of the developed aptasensor by checking its response towards common interfering mycotoxins and found negligible response. Similarly, real sample analysis was also performed using spiked rice samples, which showed satisfactory recovery percentages. Overall, the current study has paved a way for ZnSe-nanostructure-based fluorescent sensors for the detection of different biomolecular targets.

## Figures and Tables

**Table 1:** Zeta potential values of ZnSe nanostructures before and after the addition of Rhd-B.

Sr. No.	Zeta (mV) ZnSe NP	Zeta (mV) ZnSe NP+ Rhd-B
B6	−437	−229
B9	−95.2	−319
C6	−320	−284
C9	−409	−333
A6	−332	−366
A9	−379	−297

**Table 2:** Determination of OTA spiked in rice extract (n = 3).

Sr. No.	OTA Added (ng/L)	OTA Found (ng/L)	R.S.D %	R.E %	R %
1.	5	5.04	0.5634	0.8	100.8
2.	50	50.16	0.2259	0.32	100.32
3.	100	100.31	0.2188	0.31	100.31

R.S.D % = relative standard deviation percentage; R.E % = relative error percentage; R% = recovery percentage.

## Data Availability

Not applicable.

## Supplementary Materials

The following supporting information can be downloaded at: https://www.mdpi.com/article/10.3390/bios12100844/s1, “Instruments and reagents” section, Figure S1: Optimization of Rh-B dye concentration (10–250 ng/L) for the development of ZnSe nanostructures based aptasensor, Table S1: The crystalline size of nanoparticles calculated using Scherrer and strain by applying Williamson-Hall plot, Table S2: Comparison of some recent nanostructured based fluorescent sensors for OTA detection. References [35,39,40,41,42,43,44,45,46] are cited in Supplementary Materials.
