# Supplementary material for: Multifunctional Smart ZnSe-Nanostructure-Based Fluorescent Aptasensor for the Detection of Ochratoxin A
Source: Biosensors (Basel). 2022 Oct 8;12(10):844. doi: 10.3390/bios12100844 (PMC9599676; doi:10.3390/bios12100844)
Supplement: Supplementary file 1 [file biosensors-12-00844-s001.zip › biosensors-1928866-supplementary.pdf]

Supporting Information

Multifunctional Smart ZnSe-Nanostructure-Based Fluorescent Aptasensor for the Detection of Ochratoxin A

Muhammad Azhar Hayat Nawaz[1,2,3], Muhammad Waseem Fazal[3], Naeem Akhtar[4], Mian Hasnain Nawaz[3], Akhtar Hayat[3]*, Cong Yu[1,2]*

[1]State Key Laboratory of Electroanalytical Chemistry, Changchun Institute of Applied Chemistry, Chinese Academy of Sciences, Changchun, 130022, China.

[2]University of Science and Technology of China, Hefei, 230026, China

[3]Interdisciplinary Research Centre in Biomedical Materials (IRCBM), COMSATS University, Islamabad, Lahore Campus, Lahore, 54000, Pakistan

[4] Institute of Chemical Sciences, Bahauddin Zakariya University (BZU), Multan 60800, Pakistan

*Corresponding authors

E-mail: akhtarhayat@cuilahore.edu.pk, congyu@ciac.ac.cn

1. Instruments and Reagents

Zinc Nitrate, Selenium tetrachloride, hydrazine dihydrochloride, ethanol, ethylene glycol, OTA and amino modified OTA aptamer were purchased from Merck KGaA, Darmstadt, Germany.The characteristic sequence of the amino modified OTA aptamer is:

5′-[AmC6] GAT-CGG-GTG-TGG-GTG-GCG-TAA-AGG-GAG-CAT-CGG-ACA. Rhodamine-B fluorescence organic dye was purchased from AVONCHEM (Cheshire UK), while acetonitrile was obtained from BDH chemicals and phosphate buffer saline tablets (PBSpH-7.4) were purchased from bio- world (bioplus research chemicals). For the preparation of solution each buffer tablets were dissolved in 100ml of deionized water which received from ELGA Pure lab® Ultra water deionizer.Rice extract was prepared by dissolving 1gram of fine rice powder to acetonitrile and deionized water in ratio (6/4-v/v). PBS (pH-7.4) was used to prepare all solutions.

Instruments used during this scientific work were Nicolet™ iS50 FTIR Spectrometer (USA) to record FTIR spectra of ZnSe in the range of 400-4000 cm^{-1}. X-ray diffraction (XRD) was measured by Rigaku, Miniflex-II (Japan) by means of Cu Kα (with a scan angle: 5°-80°, at 40 kV, 40 mA, and (2q). The surface morphological analyses of the synthesized materials were analyzed using scanning electron microscopy (SEM) TESCAN VEGA3 LMU (Czech Republic). Raman and PL spectra was obtained using in Via Raman Microscope by RENISHAW (UK) with excitation laser of 514nm (laser power: 100%, grating: 1800 I/mm) and laser exposure time of 10s. Ultrasonication has been done by placing of 1.5 ml Eppendorf keeping the temperature below 40 ºC in Elma-sonic Ultrasonicator. ZetasizerNanoZS, Malvern Instruments, (USA) was used to measure zeta potentials of the synthesized nanostructures. BET surface area was found using Quantachrome, AutosorbiQ-C-MP surface analyzer (Anton Paar Quanta Tec Inc. USA). Fluoroskan Ascent version FL 2.6 from M/S Thermo Life Sciences, (CergyPontoise, France) was used for fluorescence measurements of ZnSe nanostructures.

Table S1. The crystalline size of nanoparticles calculated by using Scherrer and strain by applying Williamson-Hall plot

Sample	Parameter K	Lambda	**Peak position** 2( )	**FWHM**	Crystalline size (nm)	Scherrer Average Size(nm)	**X-axis** 4Sin( )	**Y-axis** Cos( )	Intercept	slope m	Microstrain	William Hall D (nm)
A6	0.9	0.154	31.862	0.5104	16.1777	16.692	1.0979	0.008	0.0068	0.0011	1.1	20.38
			65.274	4.2E-1	22.4182		2.1572	0.006				
			72.616	0.858	11.4803		2.3685	0.012				
A9	0.9	0.154	31.610	0.4045	20.4015	21.142	1.0894	0.006	0.0123	-0.0027	-2.7	11.26
			45.055	0.7963	10.7953		1.5325	0.012				
			65.356	0.3040	31.0326		2.1596	0.004				
			72.898	0.4419	22.3393		2.3764	0.006				
B6	0.9	0.154	31.815	0.4912	16.8076	16.482	1.0963	0.008	0.0094	-0.0005	-0.5	14.74
			45.603	0.5929	14.5293		1.5501	0.009				
			64.825	0.5394	17.4375		2.1440	0.007				
			72.080	0.5725	17.1544		2.3534	0.008				
B9	0.9	0.154	36.321	0.4998	16.7187	17.912	1.2467	0.008	0.0092	-0.0008	-0.8	15.06
			62.858	0.5017	18.5502		2.0857	0.007				
			72.926	0.5347	18.4661		2.3772	0.007				
C6	0.9	0.154	27.136	0.2298	35.5491	30.06	0.9384	0.0039	0.0048	-6E-05	-0.06	28.87
			31.647	0.3261	25.3104		1.0907	0.0055				
			36.150	0.3187	26.2085		1.2410	0.0053				
			45.141	0.2632	32.6744		1.5353	0.0042				
			53.520	0.2849	31.2071		1.8010	0.0044				
			65.815	0.3073	30.7737		2.1731	0.0045				
			72.609	0.3434	28.6956		2.3683	0.0048				
C9	0.9	0.154	45.055	0.3525	24.3831	23.912	1.53253	0.0057	0.0047	0.0007	0.7	29.48
			36.041	0.3753	22.2505		1.2374	0.0062				
			27.036	0.2975	27.45		0.9350	0.005				
			31.550	0.3397	24.292		1.0874	0.0057				
			34.196	0.3187	26.0632		1.1760	0.0053				
			53.435	0.3800	23.3902		1.7983	0.0059				
			65.76	0.4506	20.9838		2.1715	0.0066				
			72.541	0.4380	22.487		2.3664	0.0062				

Scherer equation

$$d = k.\lambda / \beta.Cos \qquad \text{(Equation S1)}$$

Scheme 1. d = Crystallite size (nm).

β (the peak width of the diffraction peak or full width at the half maximum: FWHM)

Cos θ = (θ is the half of diffraction angle).

Figure S1. Optimization of Rh-B dye concentration (10-250 ng. L^{-1}) for the development ofZnSe nanostructures based aptasensor

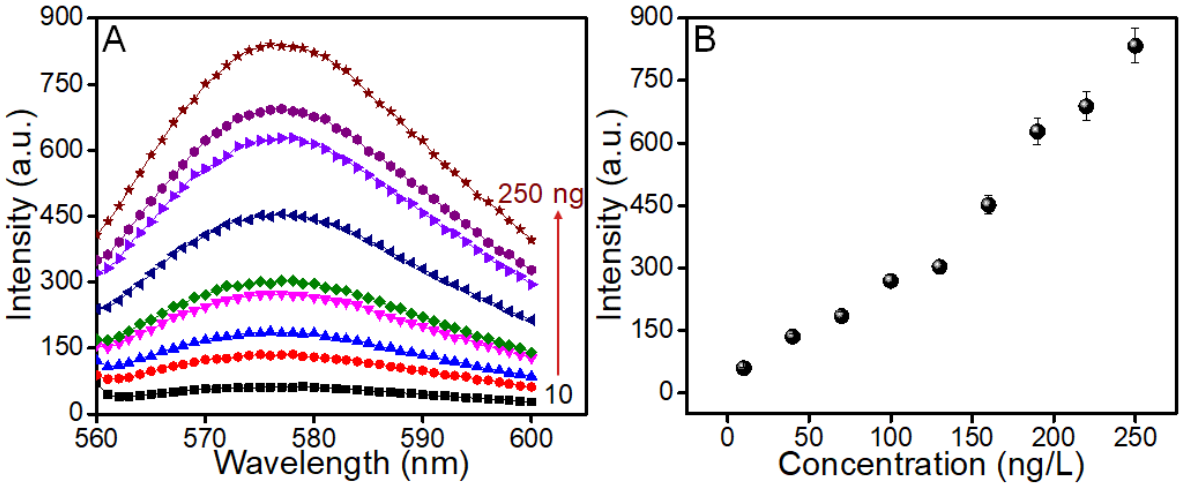

Table S2. Comparison of some recent nanostructured based fluorescent sensors for OTA detection.

Method/Material	Linear range (ng/L)	Detection Limit (ng/L)	Reference
NiO nanostructures	0.5 – 200	0.49	[35]
cerium oxide nanoparticles (nanoceria) and graphene quantum dots (GQDs)	$1\times10^1 – 2\times10^4$	2.5	[39]
PVP-coated gold nanoparticles	$4\times10^3 – 4\times10^5$	2×10^3	[40]
Titanium Dioxide Nanoparticles (TiO2)	$6\times10^2 – 4\times10^5$	6×10^2	[41]
MoS2 nanosheets	$1\times10^3 – 1\times10^6$	1×10^3	[42]
Gold and Silica nanoparticles	$4.5\times10^1 – 6.12\times10^1$	6.05×10^2	[43]
Silver nanoparticle (AgNP)	$80 – 5\times10^3$	60	[44]
Gold nanoparticles (AuNPs)	$1\times10^4 – 1.2\times10^5$	9.1×10^3	[45]
cadmium sulfide semiconductor	$2\times10^1 – 2\times10^3$	4.8	[46]

quantum dots (CdS QDs)			
ZnSe nanostructures	0.1 – 200	0.072	This work

References (The order numbers of references are consistent with that in the Manuscript)

35. Khan, A.; Nawaz, M.A.H.; Akhtar, N.; Raza, R.; Yu, C.; Andreescu, S.; Hayat, A. Morphology controlled NiO nanostructures as fluorescent quenchers for highly sensitive aptamer-based FRET detection of ochratoxin A. *Appl. Surf. Sci.* **2021**, *566*, 150647.
39. Tian, J.; Wei, W.; Wang, J.; Ji, S.; Chen, G.; Lu, J. Fluorescence resonance energy transfer aptasensor between nanoceria and graphene quantum dots for the determination of ochratoxin A. *Anal. Chim. Acta* **2018**, *1000*, 265–272
40. Lv L, Jin Y, Kang X, Zhao Y, Cui C, Guo Z. PVP-coated gold nanoparticles for the selective determination of ochratoxin A via quenching fluorescence of the free aptamer. *Food chemistry.* **2018** *30;249:45-50*.
41. Sharma A, Hayat A, Mishra RK, Catanante G, Bhand S, Marty JL. Titanium dioxide nanoparticles (TiO2) quenching based aptasensing platform: Application to ochratoxin A detection. *Toxins*. **2015** *22;7(9):3771-84*.
42. Lu Z, Chen X, Hu W. A fluorescence aptasensor based on semiconductor quantum dots and MoS2 nanosheets for ochratoxin A detection. *Sensors and Actuators B: Chemical*. **2017** *1;246:61-7*.
43. Taghdisi SM, Danesh NM, Beheshti HR, Ramezani M, Abnous K. A novel fluorescent aptasensor based on gold and silica nanoparticles for the ultrasensitive detection of ochratoxin A. *Nanoscale*. **2016**;*8(6):3439-46*.
44. Jiang H, Li X, Xiong Y, Pei K, Nie L, Xiong Y. Silver nanoparticle-based fluorescence-quenching lateral flow immunoassay for sensitive detection of ochratoxin A in grape juice and wine. *Toxins*. **2017** *28;9(3):83*.
45. Lv X, Zhang Y, Liu G, Du L, Wang S. Aptamer-based fluorescent detection of ochratoxin A by quenching of gold nanoparticles. *Rsc Advances*. **2017**;*7(27):16290-4*.
46. Wei M, Wang C, Xu E, Chen J, Xu X, Wei W, Liu S. A simple and sensitive electrochemiluminescence aptasensor for determination of ochratoxin A based on a nicking endonuclease-powered DNA walking machine. *Food chemistry*. **2019** *1;282:141-6*.
